# Study of the Role of siRNA Mediated Promoter Methylation in DNMT3B Knockdown and Alteration of Promoter Methylation of CDH1, GSTP1 Genes in MDA-MB -453 Cell Line

**Published:** 2017

**Authors:** Mojgan Naghitorabi, Hamid Mir Mohammad Sadeghi, Javad Mohammadi Asl, Mohammad Rabbani, Abbas Jafarian-Dehkordi

**Affiliations:** a *Department of Pharmacognosy, School of Pharmacy, Ahvaz Jundishapur University of Medical sciences, Ahvaz, Iran. *; b *Department of Pharmaceutical Biotechnology, School of Pharmacy and Pharmaceutical Sciences, Isfahan University of Medical Sciences, Isfahan, Iran.*; c *Cancer, Petroleum and Environmental Pollutants Research Center, Ahvaz Jundishapur University of Medical sciences, Ahvaz, Iran. *; d *Department of Pharmacology and Toxicology, School of Pharmacy and Pharmaceutical Sciences, Isfahan University of Medical Sciences, Isfahan, Iran.*

**Keywords:** Epigenetic, Promoter methylation, Gene silencing, siRNA, DNMT3B

## Abstract

Promoter methylation is one of the main epigenetic mechanisms that leads to the inactivation of tumor suppressor genes during carcinogenesis. Due to the reversible nature of DNA methylation, many studies have been performed to correct theses epigenetic defects by inhibiting DNA methyltransferases (DNMTs). In this case novel therapeutics especially siRNA oligonucleotides have been used to specifically knock down the DNMTs at mRNA level. Also many studies have focused on transcriptional gene silencing in mammalian cells via siRNA mediated promoter methylation. The present study was designed to assess the role of siRNA mediated promoter methylation in DNMT3B knockdown and alteration of promoter methylation of Cadherin-1 (CDH1), Glutathione S-Transferase Pi 1(GSTP1), and DNMT3B genes in MDA-MB-453 cell line.

MDA-MB-453 cells were transfected with siDNMT targeting DNMT3B promoter and harvested at 24 and 48 h post transfection to monitor gene silencing and promoter methylation respectively. DNMT3B expression was monitored by quantitative RT-PCR method. Promoter methylation was quantitatively evaluated using differential high resolution melting analysis.

A non-significant 20% reduction in DNMT3B mRNA level was shown only after first transfection with siDNMT, which was not reproducible. Promoter methylation levels of DNMT3B, CDH1, and GSTP1 were detected at about 15%, 70% and 10% respectively, in the MDA-MB-453 cell line, with no significant change after transfection.

Our results indicated that siDNMT sequence were not able to affect promoter methylation and silencing of DNMT3B in MDA-MB-453 cells. However, quantitation of methylation confirmed a hypermethylated phenotype at CDH1 and GSTP1 promoters as well as a differential methylation pattern at DNMT3B promoter in breast cancer.

## Introduction

Epigenetic silencing is amongst the main mechanisms that lead to the inactivation of tumor suppressor genes (TSGs) during cancer development and progression. It usually occurs through hypermethylation of the CpG islands located at the promoter regions of TSGs. This change is the result of a complex interplay of at least three different DNA methyltransferases (DNMTs), including DNMT1, DNMT3A, and DNMT3B in mammalian cell (1-3).

More interestingly, overexpression of DNMTs, especially DNMT1 and DNMT3B has been observed in many human cancers, such as sporadic breast carcinomas, hepatocellular carcinoma, and myelogenous leukemia (1-9). Several studies have reported that the increased expression of the DNMTs can result in hypermethylation of specific TSGs and poorer prognosis of cancer (1, 4-6). However, some reports have shown that there is no correlation between DNA hypermethylation of the target genes and overexpression of DNMTs (2, 7).

In addition, overexpression of DNMTs in cancer may be related to hypomethylation of their promoter regions, as it has been shown by Rajendran *et al*. (10). In our previous study we also showed that DNMT3B promoter in breast cancer patients tends to be hypomethylated (11). Although, a study by Zhu *et al.* has shown a low methylation level at the promoter region of DNMT3B in both normal and tumor samples with no significant difference between these groups (8). Drini and coworkers also showed that DNMT genes in the normal and hyperplastic polyposis tissues were similarly unmethylated (12). 

Because of the reversible nature of DNA methylation, many studies have been performed on the use of chemical compounds such as 5-Aza-cytidine to correct these epigenetic defects through demethylating DNA at the promoter region of TSGs. Despite the success of these epigenetic drugs, toxicity remains an important issue for epigenetic therapies (8, 13-16). Therefore, many researchers have focused on novel therapeutic agents including antisense and small interfering RNA (siRNA) oligonucleotides to specifically target and inhibit DNMTs (17-18). Several studies have been successfully conducted to suppress the expression of DNMTs especially DNMT3B in cancer cell lines (8, 13-14). Some of these studies have shown that siRNA mediated knockdown of DNMTs can lead to a decrease in promoter methylation of some specificTSGs and thus inducing their expression in cancer cell lines (7, 13, 19-21). However, in some cases, the reduction in promoter methylation of some other TSGs has not been reported after siRNA mediated knockdown of DNMTs (8, 13-14, 19). 

Additionally, siRNAs can induce DNA methylation and transcriptional silencing of homologous promoters in some organisms. Several studies have been successfully performed on transcriptional gene silencing (TGS) in human cells using siRNA constructs that target the homologous promoter sequence in DNA and result in promoter methylation. Silencing at the transcriptional level can provide stable and long-term knock down of the gene of interest (22-25). 

In the present study we attempted to knockdown the expression of DNMT3B at the transcriptional level, via siRNA mediated promoter methylation in the breast cancer cell line, MDA-MB-453. This is a hypermethylator cell line which exhibits high levels of total DNMT activity, overexpression of DNMT3B, and concurrent silencing of multiple methylation sensitive genes (26). The aim of our study was to assess the effect of siRNA mediated TGS on DNMT3B gene knockdown and reversing the promoter methylation of Cadherin-1 (CDH1), Glutathione S-Transferase Pi 1 (GSTP1), and DNMT3B genes in MDA-MB-453 cells.

## Experimental

*Materials and methods*

*Cell lines and culture conditions*

Human breast cancer cell line MDA-MB-453 (NCBI # C214) was obtained from Pasteur Institute of Iran (Tehran, Iran). Breast cancer cells were grown in Roswell Park Memorial Institute (RPMI) 1640 medium (Bioidea, Iran) containing 10% fetal bovine serum (Gibco, UK), and 1% Penicillin–Streptomycin (Gibco, UK). Cultures were split 1 in 3 twice weekly unless otherwise specified for transfection assays. Cells were maintained at 37°C in the presence of 5% CO_2_. The cell viability was determined by standard Trypan blue dye exclusion method.

*Designing siRNA*

An siRNA duplex specifically targeting the promoter region of human DNMT3B gene (HGNC: 2979) at -415 to -396 bases upstream of the transcription start site was designed using siDESIGN Center tool (Dharmacon, USA). A blast search indicated that the designed siRNA specifically targeted the DNMT3B gene but not the DNMT3B mRNA variants. The sequence of DNMT3B-siRNA duplex was as follows: sense strand (siDNMT-S1): 5’- GGG UUA AAG CGG AGA CUC UTT-3’, antisense strand (siDNMT-AS1): 5’- AGA GUC UCC GCU UUA ACC CTT -3’. The siRNA duplex against DNMT3B gene promoter (siDNMT) and also a random negative control siRNA duplex (siNC) were commercially synthesized (Synthesisgene, China). 

**Figure 1:**
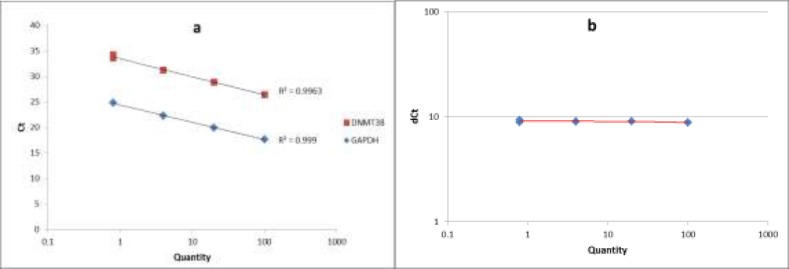
The standard curve and the validation experiment for relative quantitation of DNMT3B transcript to GAPDH (a) Relative standard curve was generated via amplifying serial five-fold dilutions (i.e. 100, 20, 4 and 0.8) of an unknown sample as the PCR template; (b) Validation experiment was performed by graphing ∆C_T_ values against relative quantities

**Figure 2:**
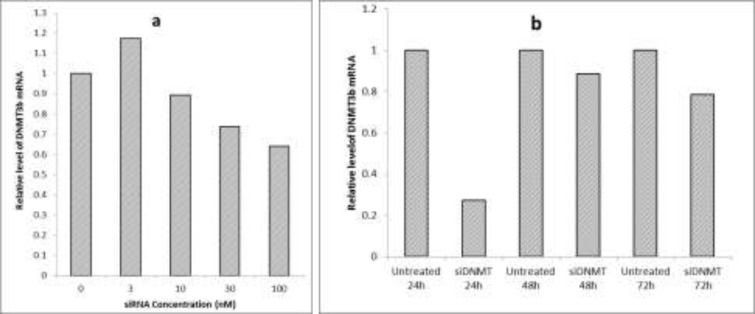
Optimization of siRNA transfection conditions (a) The effect of different concentrations of siDNMT on DNMT3B mRNA expression in MDA-MB-453 cell line after 72 h; (b) The effect of 30 nM siDNMT on DNMT3B mRNA expression in MDA-MB-453 cell line at different time points. Optimization experiments were performed once

**Figure 3:**
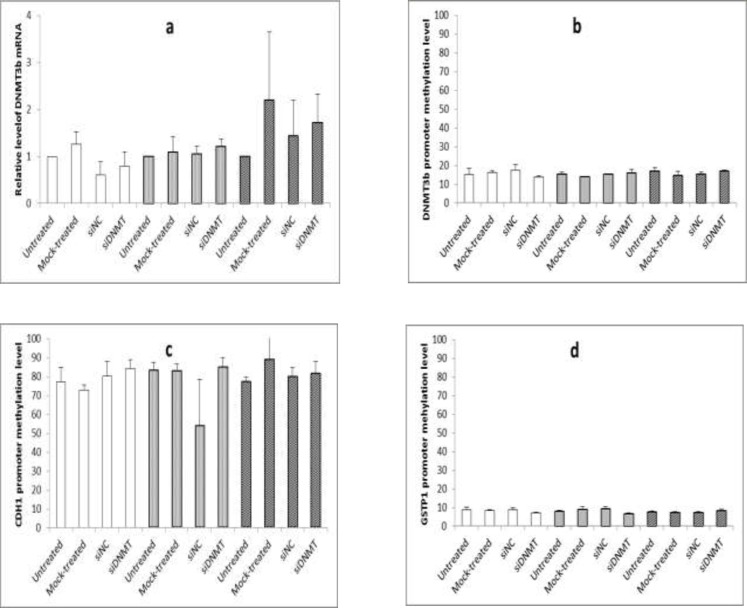
The effect of long term siDNMT transfection on the expression level of DNMT3B mRNA after 24 h (a) and the promoter methylation level of DNMT3B (b), CDH1 (c) and GSTP1 (d) after 48 h in MDA-MB-453 cell line. The siDNMT, siNC, mock-treated and untreated represents cells treated with siRNA against DNMT3B promoter, negative control siRNA, transfection reagent and culture medium, respectively; the last three were used as the negative controls. Open bars represent samples from the first transfection, gray bars represent samples from the second transfection and shaded bars represent samples from the third transfection. Values are expressed as mean ± SEM from 3 separate experiments and differences were considered significant at P < 0.05

*Transfection of siRNA*For transfection of siRNA, 24 h prior to the treatment, cells were plated at a density of 5 x 10^4^ cells per well of a 24-well plate in 0.5 mL of appropriate culture medium containing serum and antibiotics. Cells were incubated under normal growth conditions (typically 37 °C and 5% CO_2_) to reach a confluency of 70% by next day. On the day of transfection, the required amount of the siRNA (siDNMT or siNC) was diluted in 100 μL culture medium without serum and antibiotics. As per manufacturer’s instructions, 3 μL of HiPerFect Transfection Reagent (Qiagen, Germany) was added to the diluted siRNA and mixed by vortexing. The mixture was incubated for 10 min at room temperature to allow the formation of transfection complex. The complex was then added drop-wise onto the cells while gently swirling the plate to ensure uniform distribution of the transfection complex. The cells were incubated with the transfection complex under their normal growth conditions and monitored for gene silencing after the appropriate time.

In order to optimize the siRNA transfection, various concentrations of siDNMT were tested on the breast cancer MDA-MB-453 cells. This was performed by diluting equivalent volumes of a 2 µM siRNA stock solution in 100 μL culture medium without serum and antibiotics, so that it gives the final siRNA concentrations of 3, 10, 30 and 100 nM after adding transfection complexes to cells in next steps. The efficiency of gene silencing was monitored 72 h post transfection and compared to the untreated control. After optimization, the final concentration of siRNA was adjusted at 30nM. To monitor gene silencing at the appropriate time point, MDA-MB-453 cells were transfected with 30nM siDNMT and the efficiencies of gene silencing was monitored at different time points (i.e. 24, 48 and 72 h after transfection), compared to their untreated controls. 

For long term transfection, transfected cells were maintained under normal growth conditions. Forty eight h post transfection, when the cells became confluent, they were split 1: 2 into fresh plates and were re-transfected on the next day (i.e. 3 and 6 d after first transfection). Cells were harvested at 24 h post transfection (i.e, on days 1, 4, and 7) to monitor the gene silencing and at 48 h post transfection (i.e, on days 2, 5, and 8) to analyze the DNA methylation. In all transfection experiments, the silencing efficiency of siDNMT in MDA-MB-453 cells was tested against untreated, mock-treated, and siNC treated cells. Experiments were carried out thrice independently.

*RNA isolation and reverse transcription*

Total RNA was isolated from breast cancer cells (harvested 24 h post transfection) using RNeasy Mini kit (Qiagen, Germany) as recommended by the manufacturer. RNA concentration was quantified by Nano Spec Cube instrument (German Precision Nanolytic, Germany) and 1 µg of total RNA was used for synthesis of first strand complementary DNA (cDNA). Reverse transcription was performed by High Capacity cDNA Reverse Transcription kit (Applied Biosystems, USA) according to the manufacturer’s instructions using random primers supplied in the kit . The cDNAtemplates were then subjected to Quantitative real-time polymerase chain reaction (Q-PCR) for analyzing the gene silencing.

*Quantitative real-time polymerase chain reaction (Q-PCR)*

Q-PCR was performed to assess transcript expression of DNMT3B relative to glyceraldehyde-3-phosphate-dehydrogenase (GAPDH) as the endogenous control. Two microliters of cDNA were used in a 20 µL reaction with 1X SYBR Green PCR Master Mix (Life Technologies, USA) and 0.25 µM of each forward and reverse primers. The following primers were used: DNMT3BF162: 5’- AGA TCA AGC TCG CGA CTC TC -3’, DNMT3BR162: 5’- GAC AGC TGG GCT TTC TGA AC-3’, GAPDHF113: 5’- CTC AAC TAC ATG GTT TAC A -3’, and GAPDHR113: 5’- AAG ATG GTG ATG GGA TTT -3’. The reaction was performed in an ABI StepOne Real-Time PCR Systems (Applied Biosystems, USA) using the following conditions: a denaturation step lasting 10 min at 95 °C, followed by 45 repeats of the following cycle: 95 °C for 15 s, annealing at 53 °C for 15 s and extension at 72 °C for 20 s. after the 45th cycle, an optional denaturation and renaturation step was carried out for 15 s at 95 °C and 1 min at 60 °C, followed by a melt curve step ramping from 60 °C to 85 °C rising 1 °C per s. Relative quantitation was performed using comparative C_T_ (∆∆C_T_) method. Data were normalized to GAPDH expression as the endogenous control and untreated sample as the reference sample. ROX dye was used as the passive reference. Also no template control (NTC) was included as the negative control. Each sample was analyzed in duplicate.

The quality of PCR products was checked by melting curve analysis. To assess the PCR efficiencies in amplification of both DNMT3B and GAPDH genes, a relative standard curve experiment was performed. The standard curve was generated via amplifying serial five-fold dilutions (i.e. 100, 20, 4 and 0.8) of an unknown sample as the PCR template. Finally we performed a validation experiment by graphing ∆C_T_ values against relative quantities, to assess the validity of ∆∆C_T_ method in our study.

*DNA extraction and bisulfite modification*

Genomic DNA was extracted from breast cancer cells (harvested 48 h post transfection) with Blood-Animal-Plant DNA Preparation kit (Jena Bioscience, Germany) as per the manufacturer’s instructions. After determining the DNA concentration of samples by Nano Spec Cube instrument (German Precision Nanolytic, Germany), Sodium bisulfite modification of DNA was performed using Epitect Bisulfite kit (Qiagen, Germany) according to the manufacturer’s instructions. EpiTect Control DNA Unmethylated and Unconverted (Qiagen, Germany) was used as the control for bisulfite treatment method. Bisulfite treated DNA was subjected to DNA methylation analysis.

*DNA methylation analysis*

DNA methylation analysis was performed on bisulfite treated DNA samples at the promoter region of DNMT3B, CDH1 and GSTP1 genes using quantitative method of differential high resolution melting analysis (DHRMA). A Rotor-Gene 6000 (Corbett Research, Australia) was utilized for PCR amplification and subsequent HRM analysis. Details of the DHRMA method for methylation analysis including the primer pair, PCR and HRM conditions and the data analysis have been described previously for DNMT3B (11). The DHRMA method for CDH1 and GSTP1 genes was performed by a similar protocol for DNMT3B gene, except using specific primer pairs, PCR, and HRM conditions.

For CDH1 DHRMA, the primer sequences specified for CDH1 gene (HGNC: 1748) were as follows: meCDH1F120: 5’-GGT TGG GTA ATA TAG GGA GAT ATA G-3’ and meCDH1R120:5’-AAA ATA CAA ATA CAC ACC ACC AC-3’. PCR was performed in 20 μL volume containing: 1X Epitect HRM PCR Master Mix (Qiagen, Germany), 750 nM of each primer, and a 100 ng bisulfite-converted DNA template. The touchdown amplification program was, a 5 min hold at 95 °C, followed by 55 cycles, including 10 s of denaturation at 95 °C, 30 s of annealing at 55 °C, decreasing 0.2 °C per cycle to 50 °C, and then 10 s extension at 72 °C. An optional denaturation and renaturation step were performed for 30 s at 95 °C and 30 s at 50 °C, followed by HRM step ramping from 60 °C to 85 °C, rising 0.1 °C every 2 s.

The GSTP1 DHRMA was carried out using a specific primer pair for GSTP1 (HGNC: 4638) with following sequences: meGSTP1F104: 5’-TTTAGAATTTTAAATAAAAGTTGGA-3’ and me GSTP1R104:5’ACTCCTAACCTTAAATAATCTACAC-3’. PCR was performed in 20 μL volume containing: 1X Epitect HRM PCR Master Mix (Qiagen, Germany), 750 nM of each primer and 100 ng bisulfite treated DNA template. The amplification program was 5 min at 95 °C, then 55 cycles including 10 s at 95 °C, 30 s at 55 °C, 10 s at 72 °C. An optional denaturation and renaturation step was performed for 30 s at 95 °C and 30 s at 50 °C, followed by an HRM step ramping from 60 °C to 85 °C rising 0.1 °C per 2 s. 

*Data analysis*

Data was recorded in a Microsoft excel spreadsheet and Statistical Package for the Social Sciences (SPSS) software version 16.0 (SPSS Inc, USA) was used for statistical analysis. Data analysis was performed using one way ANOVA to analyze the significance between different values. Values are expressed as mean ± SEM from at least 3 separate experiments and differences were considered significant at a P value of less than 0.05.

## Results

*Efficiency of Q-PCR in mRNA expression analysis of DNMT3B*

A relative standard curve experiment was performed to assess the PCR efficiencies of DNMT3B and GAPDH amplification. According to the standard curve, the PCR efficiencies of DNMT3B and GAPDH were 0.99. The validation experiment also confirmed the validity of ∆∆C_T_ method in our study. Figure 1 shows the standard curve and the validation experiment for relative quantitation of DNMT3B transcript to GAPDH.

*Optimization of siRNA transfection conditions*

The breast cancer MDA-MB-453 cells were transfected with various concentrations of siDNMT (i.e. 3, 10, 30 and 100 nM) and the efficiency of gene knock down was monitored 72 h post transfection. Figure 2a shows the effect of different concentrations of siDNMT on DNMT3B mRNA expression. As the graph shows, increasing the siDNMT concentration decreased the DNMT3B mRNA expression. However, using higher siDNMT concentrations (100 nM) resulted in changes in the morphology and cell death in long term transfection experiment (data not shown). Therefore, the final siRNA concentration of 30 nM was used for transfection of MDA-MB-453 cells in next experiments. Also the silencing effect of siDNMT was monitored at different time points (i.e. 24, 48 and 72 h post transfection) in MDA-MB-453 cells transfected with 30nM siDNMT. As Figure 2b shows, 24 h after transfection, the silencing effect of siDNMT is higher than other time points.

*The effect of siRNA transfection on the expression of DNMT3B mRNA*

For long term transfection, MDA-MB-453 cells were transfected with siDNMT for three times (i.e, on days 0, 3, and 6) and cells were harvested 24 h post transfection to monitor gene silencing. Figure 3a shows the effect of long term siDNMT transfection on the expression level of DNMT3B mRNA in MDA-MB-453 cells after 24 h of transfection. A non-significant decrease was observed in the mRNA expression level of DNMT3B after the first transfection with siDNMT (Figure 3a, open bars). The second transfection resulted in approximately a consistent level of DNMT3B mRNA expression (Figure 3a, gray bars) and the third transfection caused a non-significant increase in the DNMT3B expression (Figure 3a, shaded bars).

*The effect of siRNA transfection on the promoter methylation level of DNMT3B, CDH1 and GSTP1 genes*

The MDA-MB-453 cells transfected with siDNMT for three times, were harvested 48 h post each transfection to analyze the DNA methylation. Figure 3b-d shows the effect of long term siDNMT transfection on the promoter methylation level of DNMT3B, CDH1, and GSTP1 in MDA-MB-453 cells after 48 h of transfection. As Figure 3b shows the methylation level at the DNMT3B promoter region is about 15% in the MDA-MB-453 cell line. No significant change in the DNMT3B promoter methylation was shown after siRNA transfections of the MDA-MB-453 cells.

For CDH1 gene, the promoter region was hypermethylated in the MDA-MB-453 cell line, with the methylation levels being above 70%. As Figure 3c shows the siRNA transfection had no significant effect on the CDH1 promoter methylation in the MDA-MB-453 cells. Also the methylation level of GSTP1 promoter was below 10% in the MDA-MB-453 cell line and no significant difference in the DNMT3B promoter methylation was observed after siRNA transfections (as shown in Figure 3d).

## Discussion

The aim of the present study was to assess the effect of siRNA mediated promoter methylation in DNMT3B knock down and quantitatively evaluate the promoter methylation of CDH1, GSTP1, and DNMT3B genes in MDA-MB -453 cell line after transfection with siRNA. We used human breast cancer cell line MDA-MB-453 in our study, because according to the literature it is a hypemethylator cell line representing high level of DNMTs activity and overexpression of DNMT3B along with silencing of multiple methylation sensitive genes (26).

We used the final siRNA concentration of 30 nM for cell transfection, since using higher concentrations (100 nM) resulted in changes in the morphology of cells and cell death in long term transfection. Although cell death was observed as floating cells in the culture and no more specified cytotoxicity assay such as MTT or XTT assay has been performed in this study. Of course these changes were only observed in long term transfection experiments i.e., after second or third transfection with siRNA. The effect of siRNA in reduction of cell proliferation has been reported by other researchers and was attributed to enhanced apoptosis due to DNMT3B silencing (7, 19). One explanation for this phenomenon could be that DNMT3B silencing promotes the expression of proapoptotic genes such as caspase-7 and cytochrome b5, as well asnegatively acting cell cycle regulators like Rb1 and CDKN3 (19).

As per results of optimization experiments the maximum silencing effect of siDNMT (about 73% reduction in relative expression of DNMT3B gene) was observed 24 h after transfection, therefore the 24 h time point samples were used to check the silencing effect. Whereas for analyzing the methylation levels samples were collected at 48 h time point. Although silencing effect of siDNMT did not last for a long time, unlike to what we expected from methylation dependent siRNAs. In the study of Pulukuri *et al*. the silencing of the target gene was quite robust and remained detectable 14 d after a single transfection of siRNA. They concluded that, in contrast with post transcriptional gene silencing that requires the continued presence of a siRNA, transcriptional gene silencing via targeting the promoter sequence allows stable, long-term silencing of the interested gene (22). 

For expression analysis we used ∆∆C_T_ method and the validation experiment confirmed the validity of ∆∆C_T_ method in our expression analysis. Although the results from the optimization experiment showed a 73-80% decrease in DNMT3B mRNA levels at 24 h post first transfection, the combined results of expression analysis did not show any significant reduction in mRNA expression level of DNMT3B after transfection of siDNMT. In general our findings suggest that the designed siDNMT construct was not able to effectively silence the DNMT3B expression. This might be due to inefficient transfection of siRNA, difficulties with successful delivery of the siRNA to the nucleus and or its access to the promoter region of DNMT3B gene, as the target site. In this case, it is highly suggested to use a fluorescence labeled control siRNA for checking the transfection efficiency and tracking the siRNA delivery into and inside the cell. Furthermore, in our study we have only tested one siRNA sequence in comparison to other studies that have used more than four siRNA or short hairpin RNA (shRNA) constructs to see a silencing effect (22-25). It has been suggested to use pools of three or more siRNA sequences per target gene for more efficient gene knockdown (27). We propose as well to test a minimum of three or four different siRNA sequences for future siRNA mediated silencing studies. 

Additionally, the results from methylation analysis of three genes did not show any significant change in methylation levels after transfection of siDNMT. These results combined with the expression analysis results might indicate that our siDNMT sequence could not have a specific effect on promoter methylation and thus silencing of DNMT3B in MDA-MB-453 cells. Although many studies have demonstrated the TGS via DNA methylation in human cells (22-25), Costanotto and colleagues were able to detect a low level of *de novo* DNA methylation and partial gene silencing, after transfecting Hela Cells with shRNAs complementary to the promoter or early transcribed regions of RASSF1A (28). Similar to our results some previous publications have also indicated that shRNAs cannot direct *de novo* methylation in murine oocytes (29), human glioblastoma (30) or colorectal cancer cell line (31). The combined results from our study and others might indicate that the rules for RNA-mediated transcriptional gene silencing and DNA methylation are not well known and this phenomenon is likely to be affected by some factors including the position or sequence composition of the target site, the gene that is being silenced, and the cell type (22, 28). Further studies using different and multiple siRNA sequences per target gene should be performed in this regard. It is suggested that fluorescently labeled siRNAs are used in future studies to be able to track them in the cell and evaluate the transfection efficiency. Also it is highly recommended to use shRNA constructs instead of siRNAs for more stable silencing effects. 

Meanwhile the methylation level at the DNMT3B promoter region was detected at about 15% in the MDA-MB-453 cell line. In our previous study using DHRMA we determined the mean methylation level of this gene which was 2.8% in breast cancer patients as compared to 3.3% in the normal marginal tissues of breast tumors. Although there was a little difference between the mean methylation level of the normal samples and that of the patients, a significant difference was shown in the methylation levels of patients. Forty eight percent of patients were classified as hypomethylated, about 38% as methylated and 14% as hypermethylated, suggesting that DNMT3B promoter in breast cancer patients tends to be hypomethylated (11). Also a study on gliomas by Rajendran *et al*. showed hypermethylation at the DNMT3B gene promoter for the normal brain tissues as well as in glioblastoma cell lines as compared to a differential DNA methylation pattern in the tumor tissue samples derived from different grades of gliomas (10).

Also CDH1 and GSTP1 promoter was determined methylated in the MDA-MB-453 cell line, with the methylation levels of more than 70% and below 10%, respectively. This is in support with the previous findings have suggested that, CDH1 and GSTP1 promoter regions tend to be methylated in breast cancer cells (32,33). 

In summary, –the results of transfection experiments indicated thatour siDNMT sequence was not able to successfully affect the promoter methylation and thus silencing of DNMT3B in MDA-MB-453 cells. However, further studies using different and multiple siRNA sequences per target should be performed in this regard. We suggest using fluorescently labeled siRNAs in future studies to be able to evaluate the transfection efficiency. Also it is highly recommended to use shRNA constructs instead of siRNAs for more stable silencing effects. Finally, quantitation of the methylation levels in MDA-MB-453 cell line confirmed a hypermethylated phenotype at CDH1 and GSTP1 promoters beside a differential methylation pattern at DNMT3B promoter in breast cancer. 
